# The long pentraxin 3: the invisible loom weaving the warp and weft of tumor destiny

**DOI:** 10.3389/fimmu.2025.1705051

**Published:** 2025-12-17

**Authors:** Tianyi Liu, Manyu Li, Yuxi Cheng, Jialin Zou, Fengying Liu, Jie Dong, Cheng Qiu, Xuenan Peng, Deyang Kong, Zhifan Li, Xinyu Wang, Lanyu Li

**Affiliations:** 1 Department of Hepatobiliary Surgery, National Cancer Center/National Clinical Research Center for Cancer/Cancer Hospital, Chinese Academy of Medical Sciences and Peking Union Medical College, Beijing, China; 2 Department of Medical Oncology, National Cancer Center/National Clinical Research Center for Cancer/Cancer Hospital, Chinese Academy of Medical Sciences and Peking Union Medical College, Beijing, China; 3 Department of Gastroenterology, Qilu Hospital of Shandong University, Jinan, Shandong, China; 4 Cheeloo College of Medicine, Shandong University, Jinan, Shandong, China; 5 The First Clinical Medical College of Lanzhou University, Lanzhou, Gansu, China; 6 State Key Laboratory of Reproductive Medicine and Offspring Health, Center for Reproductive Medicine, Institute of Women, Children and Reproductive Health, Shandong University, Jinan, Shandong, China; 7 Department of Obstetrics and Gynecology, Central Hospital Affiliated to Shandong First Medical University, Jinan, China; 8 Department of Orthopedic Surgery, Peking Union Medical College Hospital, Peking Union Medical College and Chinese Academy of Medical Sciences, Beijing, China; 9 Department of Colorectal Surgery, National Cancer Center/National Clinical Research Center for Cancer/Cancer Hospital, Chinese Academy of Medical Sciences and Peking Union Medical College, Beijing, China; 10 State Key Laboratory of Molecular Oncology, National Cancer Center/National Clinical Research Center for Cancer/Cancer Hospital, Chinese Academy of Medical Sciences and Peking Union Medical College, Beijing, China; 11 Cardiometabolic Center, National Center for Cardiovascular Diseases, Fuwai Hospital, Chinese Academy of Medical Sciences and Peking Union Medical College, Beijing, China; 12 Department of Anesthesiology, National Center for Orthopaedics, Beijing Jishuitan Hospital, Capital Medical University, Beijing, China

**Keywords:** pentraxins, PTX3, cancer, biomarker, epigenetic regulation, inflammation, infection, complement

## Abstract

Pentraxins, which constitute a family of evolutionarily conserved pattern recognition molecules, are categorized into short and long branches. The long pentraxin 3 (PTX3) is a key member of the long pentraxin subfamily, while the C-reactive protein and serum amyloid P represent the short pentraxins. All pentraxins share a highly conserved C-terminal motif, an 8-amino acid sequence known as the pentraxin signature. PTX3 can be produced by a wide range of cell types, including immune cells such as dendritic cells, monocytes, and macrophages, as well as various non-immune cells, underscoring its pleiotropic roles in multiple pathophysiological processes. These include inflammation, infection, tissue repair, female fertility, and cancer. Although PTX3 engages commonly recognized signaling pathways, such as TNF-α, NF-κB, FGF, and PI3K/AKT, it can exert paradoxical effects in different cellular contexts, either promoting or inhibiting the proliferation, migration, invasion, and metastasis of cancer cells. This review provides a comprehensive overview of the multifaceted roles of PTX3 in various cancers, while also summarizing its functions in other physiological or pathological contexts. Furthermore, we critically examine the challenges and translational opportunities of PTX3, aiming to inform future research directions and therapeutic strategies for cancer management.

## Introduction

The long pentraxin 3 (PTX3), a protein highly conserved throughout evolution (1), was first identified in 1992 as a gene product induced by interleukin (IL)-1β or tumor necrosis factor (TNF)-α stimulation (2). Over the past few decades, PTX3, a key member of the pentraxin superfamily, has taken center stage, with accumulating evidence illuminating its versatile roles in innate immunity, inflammation, infection, tissue repair, female fertility, and cancer (3–5).

Pentraxins belong to a class of pattern recognition molecules (PRMs), which also include complement components and innate immune receptors. This superfamily comprises phylogenetically conserved proteins characterized by a specific 8-amino-acid (aa) sequence known as the “pentraxin signature” (His-x-Cys-x-Ser/Thr-Trp-x-Ser, where x represents any amino acid) at the carboxyl terminus. Based on the length of the N-terminal region, pentraxins are categorized into two branches. Short pentraxins, such as C-reactive protein (CRP) and serum amyloid P (SAP), are primarily produced by the liver in response to IL-6 and enhance pathogen phagocytosis (6). The long pentraxin branch includes PTX3, PTX4, neuronal pentraxin 1 (NP1), and NP2. PTX3 is secreted by a diverse range of cell types, including immune cells (e.g., dendritic cells, monocytes, and macrophages) and non-immune cells such as cancer cells, typically upon stimulation by pro-inflammatory mediators (7).

This review summarizes the role of PTX3 in various cancer types. Based on existing literature, we describe how PTX3 acts as a pivotal regulator governing tumor fate. We hope this review will provide a relevant basis for developing PTX3 or its regulators as potential therapeutic targets against cancer.

## The *PTX3* gene

**Figure 1:**
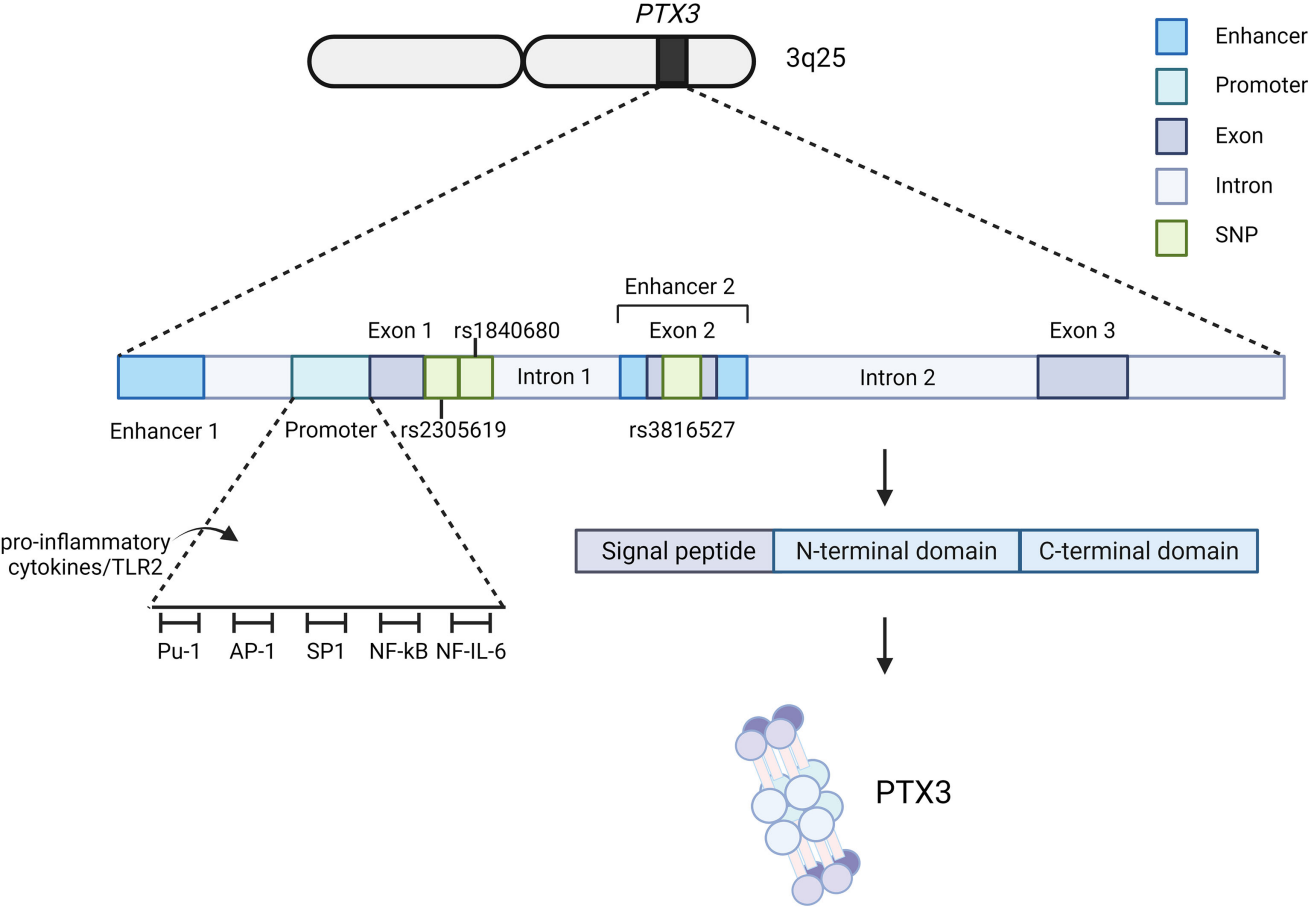
Schematic diagram of the *PTX3* gene. The *PTX3* gene is located on chromosome 3 and comprises three exons: the first encodes the signal peptide, the second the N-terminal domain, and the third the C-terminal pentraxin domain. Its promoter region harbors binding sites for multiple transcription factors. Two enhancers mediate the epigenetic regulation of *PTX3*, and three single nucleotide polymorphisms have been identified.

The human *PTX3* gene is located on chromosome 3q25 and consists of three exons. The first exon encodes a 43-aa segment containing a 17-aa signal peptide (SP); the second exon encodes a 135-aa N-terminal domain; and the third exon encodes the pentraxin domain (aa 179-381) of PTX3 (**Figure 1**) (8). The gene’s promoter region, located near the exons, contains multiple transcription factor binding sites targeted by pro-inflammatory cytokines and Toll-like receptor (TLR) agonists, leading to PTX3 induction (6). Three single nucleotide polymorphisms (SNPs) have been identified in *PTX3*: two (rs2305619 and rs1840680) are located in intronic regions, while the third (rs3816527) causes a missense mutation, substituting alanine with aspartic acid (**Figure 1**). These SNPs are associated with susceptibility to various infections, including fungal infection (9, 10), COVID-19 pneumonia (11, 12), cryptococcosis (13), leprosy (14), and acne (15). Associations with other immune disorders such as granuloma (16), graft dysfunction (17), and tumorigenesis (18) have also been reported.

## The PTX3 protein

Discovered in the 1990s, PTX3 is a secreted protein featuring a pentraxin signature domain at its C-terminus. It functions as a multimeric glycoprotein, forming an octamer composed of two tetramers. The molecular weight of PTX3 is approximately 340 kDa in its octameric form and 40 kDa in its monomeric form. Its primary sequence spans 381 amino acids, including a 17-residue SP (**Figure 1**) (2). This sequence is highly conserved, with significant similarity between human and mouse PTX3 (8, 19).

The C-terminal pentraxin-like domain of PTX3 shares homology with those of CRP and SAP, primarily due to the pentraxin signature sequence. However, unlike CRP and SAP, PTX3 lacks a calcium-binding pocket, which may explain its ability to bind C1q in a calcium-independent manner (20–23). Structural predictions based on crystallographic data from SAP and CRP suggest this domain adopts a β-jelly roll topology stabilized by intra-chain and inter-chain disulfide bonds, which are crucial for PTX3’s quaternary structure (24). A single N-glycosylation site at Asn220 within the C-terminal domain can be modified with various oligosaccharides, including fucosylated and sialylated biantennary, tri-antennary, and tetra-antennary glycans (25, 26). Evidence suggests that the specific oligosaccharide profile and sialylation level of PTX3 vary depending on the producing cell type, indicating cell-specific glycosylation patterns (25).

In contrast to the conserved C-terminus, the N-terminal domain of PTX3 does not share significant homology with other known proteins (6). Its secondary structure is predicted to form four α-helices, with three regions (aa 78-97, 109-135, and 144-170) potentially involved in forming coiled-coil structures (27, 28).

## Epigenetic regulation of PTX3

### DNA methylation

Studies have shown that PTX3 expression is suppressed in esophageal squamous cell carcinoma (ESCC) due to promoter hypermethylation (29). Similarly, in colorectal carcinoma (CRC), promoter methylation levels and CpG island methylation of the *PTX3* gene increase hierarchically from normal epithelium to adenomas and to CRC, independent of cancer stage (30). Furthermore, *PTX3* promoter methylation negatively correlates with plasma PTX3 levels in patients with coronary artery disease (31).

### Histone modification

In CRC, the repressive histone mark H3K27me3 is highly enriched at enhancer 1 of *PTX3* in stage I patients, while enhancer 2 acquires this mark in stages II and III. Interestingly, chromatin immunoprecipitation analyses revealed that treatment with the methylation inhibitor 5-AZA-dC and TNF-α elevates transcription-activating histone marks (H3K4me3, H3K27ac, and H3K9ac) at the *PTX3* promoter in CRC cells, without altering H3K27me3 levels (30, 32). Additionally, the tumor suppressor Menin upregulates PTX3 expression by enhancing H3K4me3 accumulation at its promoter in the decidua during normal pregnancy (33).

### Noncoding RNA

Bioinformatic analyses implicate that the long non-coding RNA (lncRNA) GAS5-miR-21-5p axis might be upstream of PTX3 in osteoporosis pathogenesis within osteoblastic cells (34). Other lncRNAs, such as MEG3, repress PTX3 expression by upregulating miR-770, thereby influencing placental cell growth (35). Similarly, lncRNA ROA reduces PTX3 expression to inhibit adipogenesis in mesenchymal stem cells (36).

Regarding microRNAs (miRNAs), miR-31-5p and miR-27a-3p promote cumulus expansion by enhancing Ptx3 expression during oocyte functionalization, whereas miR-351-5p and miR-503-5p exert inhibitory effects (37). In meningioma, inhibition of miR-29c increases PTX3 expression, inducing apoptosis and reducing cell viability (38). Conversely, miR-29b overexpression therapy suppresses PTX3, mitigating vascular inflammation in radiotherapy-induced vascular disease (39).

### RNA modification

N-acetyltransferase 10 enhances PTX3 mRNA stability and translation efficiency *via* ac^4^C modification, upregulating PTX3 expression and promoting synovial aggression and inflammation in rheumatoid arthritis (40). In contrast, methyltransferase-like 3 (METTL3) mediates m^6^A modification in the 3’UTR of *PTX3*, leading to transcript instability and degradation in bone marrow-derived macrophages (41). *PTX3* has also been identified as an m^6^A-related hub gene with high prognostic significance in diffuse gliomas (42).

### Chromatin remodeling

Nonsense mutations in SMARCD2, a subunit of the SWI/SNF chromatin-remodeling complex, lead to altered expression of genes involved in neutrophil maturation and function, including *PTX3* (43, 44).

## PTX3 and the complement system

**Figure 2:**
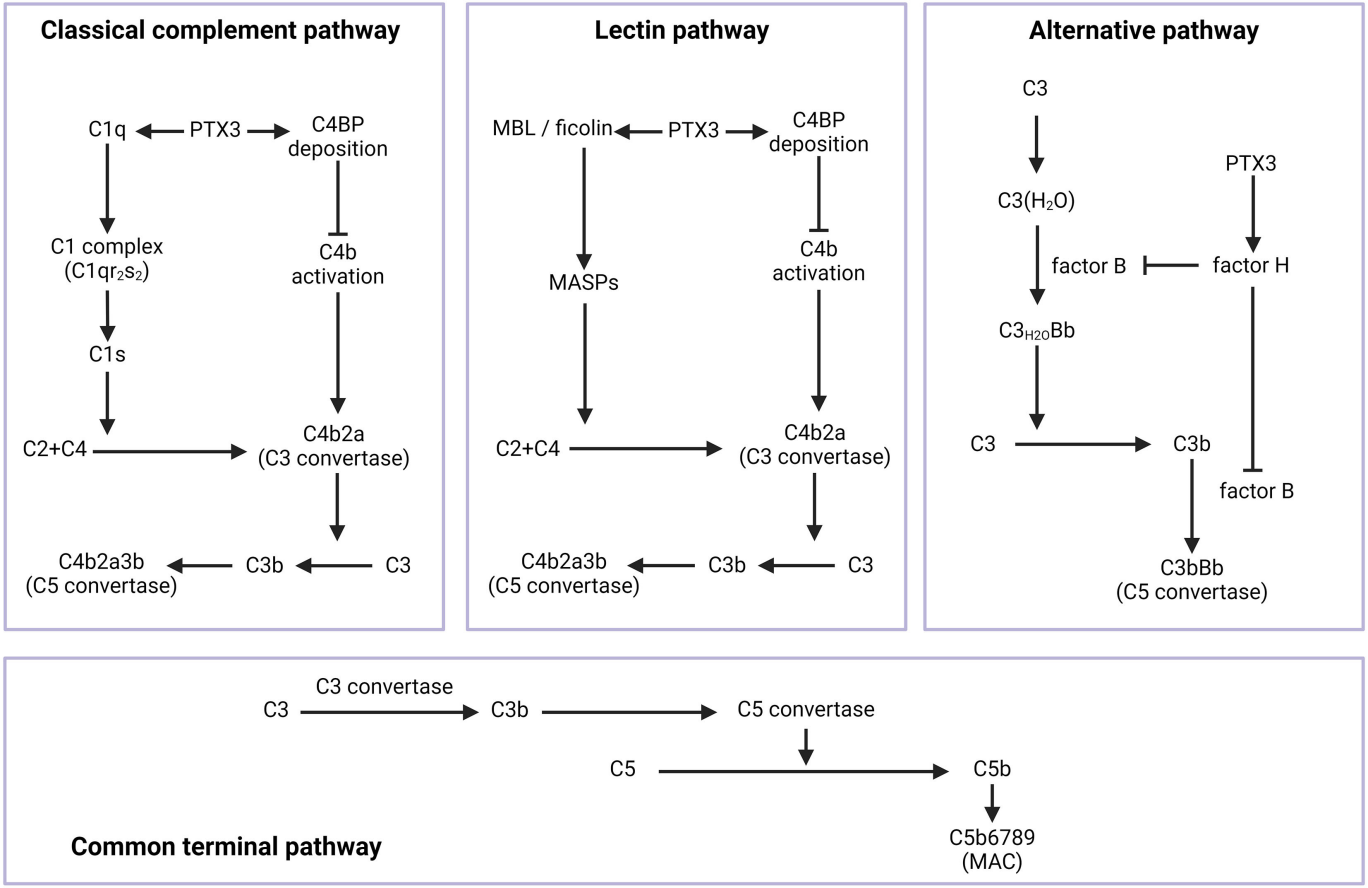
The role of PTX3 in the activation of the complement system. The complement system can be activated *via* three pathways, the classical, lectin, and alternative pathways, all converging to form C3 convertase and proceeding through a common terminal pathway. PTX3 could regulate the three pathways. In the CCP, PTX3 interacts with C1q, thereby activating the CCP. On the other hand, PTX3 can recruit C4BP deposition, inhibiting C4b activation and the formation of C3 convertase. In the LP, the binding of PTX3 to MBL or ficolin facilitates complement recruitment and the formation of C3 convertase. The C4BP deposition recruited by PTX3 is identical to the C4BP involved in the CCP. In the AP, PTX3 recruits factor H, resulting in the eventual inhibition of the C3bBb complex. Arrows denote activation, and bars denote inhibition. C4BP: C4b-binding protein; MBL: mannose-binding lectin; MASP: MBL-associated serine protease; MAC: membrane attack complex; CCP: classical complement pathway; LP: lectin pathway; AP: alternative pathway.

The complement system is a crucial component of humoral immunity (45). It can be activated *via* three pathways, the classical (CCP), lectin (LP), and alternative (AP) pathways, all converging to form C3 convertase and proceeding through a common terminal pathway (**Figure 2**) (46).

PTX3 interacts with C1q (47), thereby activating the CCP (22). Key residues involved include Lys170 in the globular head of C1q chain C (ghC) and Tyr175 in chain B (ghB) (48). PTX3 exerts dual effects on complement activation: the fluid-phase protein inhibits the cascade, while the immobilized form promotes CCP activation, enhancing C3 and C4 deposition (21).

The LP is analogous to the CCP but involves mannose-binding lectin (MBL) and ficolins instead of C1q (49). The glycosidic moiety of PTX3, particularly its terminal sialic acid, can bind to the fibrinogen-binding domain of ficolin-1 and ficolin-2, as well as the collagen-like domain of MBL (50, 51). These interactions facilitate complement recruitment on recognized cell surfaces (52). LP activation leads to the engagement of MBL-associated serine proteases (MASPs), with MASP-2 cleaving C4 and C2 to form C3 convertase, mirroring the subsequent steps of the CCP.

The AP can be initiated through spontaneous C3 hydrolysis/proteolysis (53, 54), basic pattern recognition (55), or PRM-based initiation (45, 56). While PTX3 does not directly bind to or initiate the AP, it regulates this pathway through complement regulatory proteins such as factor H (fH), C4b-binding protein (C4BP), and factor I (fI) (45), with direct interactions reported for fH and C4BP (52, 57). PTX3 recruits fH to deposited C3b, inhibiting the formation of the C3bBb complex (C3 convertase in AP) and thus restraining AP activation (58). fH inhibits the AP by displacing Bb from C3bBb and acting as a cofactor for fI-mediated C3b degradation (45). The interaction between PTX3 and fH involves two sites: one between short consensus repeat 7 (SCR 7) of fH and the glycosylated C-terminal domain of PTX3, and another between SCR 19-20 of fH and the N-terminal domain of PTX3 (24). C4BP functions similarly to fH but within the CCP and LP (45). PTX3 promotes C4BP deposition on recognized cell surfaces or the extracellular matrix (ECM), which inhibits C4b activation and prevents membrane attack complex (MAC) deposition (59). In summary, PTX3 enhances the inhibitory functions of fH and C4BP during complement activation.

## Aberrant PTX3 expression in various cancers

**Figure 3:**
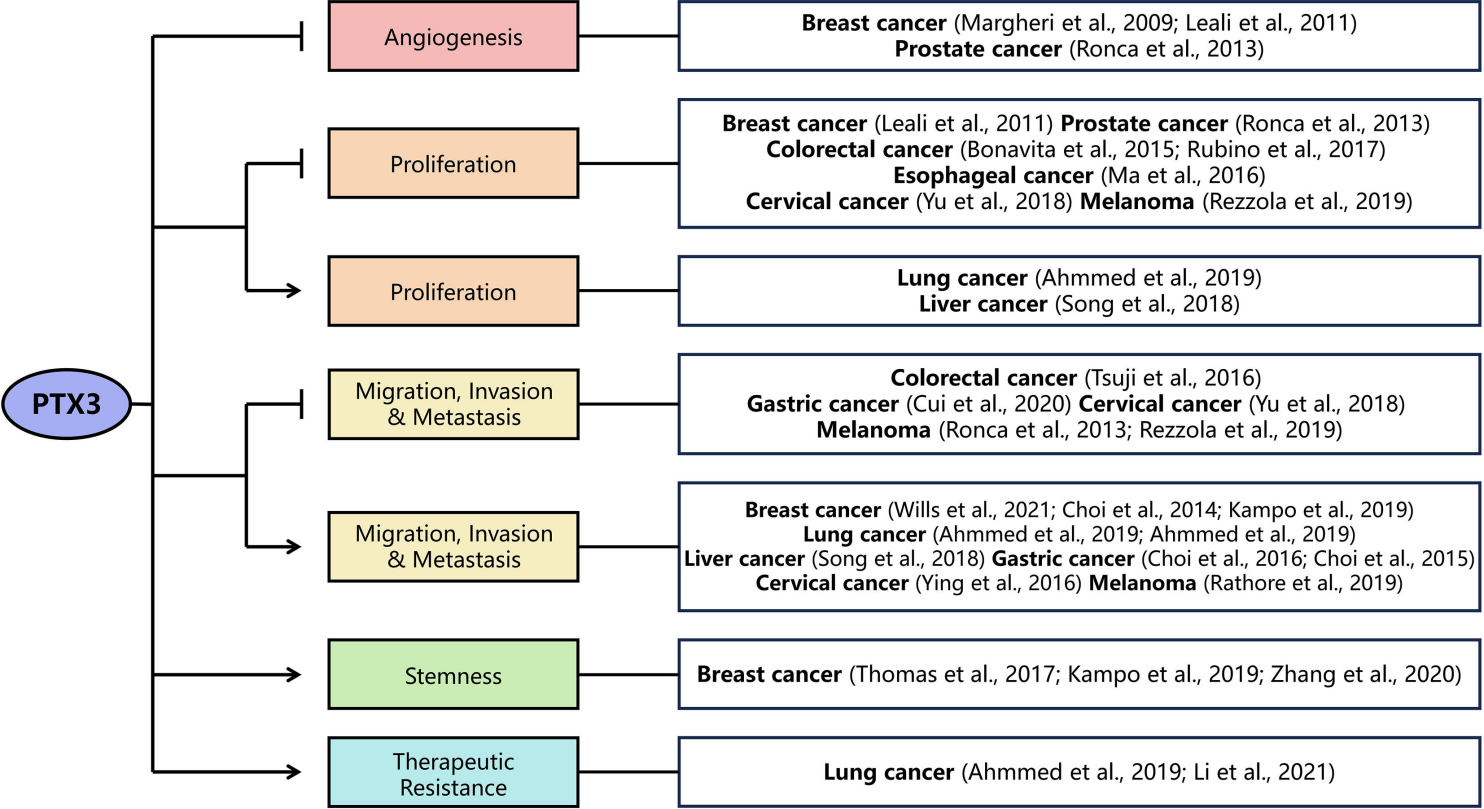
The complex role of PTX3 in various cancer types. PTX3 exhibits dual, context-dependent roles in tumorigenesis. It typically functions as a tumor suppressor by inhibiting angiogenesis, proliferation, migration, invasion, and metastasis across various cancers. Conversely, in certain cancer types, PTX3 demonstrates tumor-promoting activities by enhancing proliferation, migration, invasion, metastasis, stemness, and therapeutic resistance. Arrows denote activation, and bars denote inhibition.

The role of PTX3 in cancer development and progression has been extensively investigated. Here, we summarize its functions across various cancers (**Figure 3**) and its association with key signaling pathways, including fibroblast growth factor (FGF), nuclear factor kappa-B (NF-κB), and phosphoinositide 3-kinase (PI3K)/AKT, which are discussed in detail in subsequent sections (**Table 1**).

**Table 1:** Impact of PTX3 in various cancers.

Cancer types (role of PTX3 in tumor progression)	Main findings	Involved signaling pathways	Reference
Breast cancer (inhibitory)	The overexpression of PTX3 in mammary carcinoma cells inhibits FGF2-dependent stimulation of capillary morphogenesis.	PTX3/FGF2	(60)
Breast cancer (inhibitory)	Ptx3 inhibits dihydrotestosterone- and Fgf8b-driven angiogenesis and proliferation of androgen-regulated mouse breast tumor cells.	Ptx3/Fgf8b	(61)
Breast cancer	Peripheral PTX3 levels are increased in breast cancer patients receiving bevacizumab, compared to those receiving chemotherapy.	/	(63)
Breast cancer (promoting)	PTX3 as a small extracellular vesicle-associated protein, is negatively associated with relapse-free survival and accelerates chemotherapy-induced breast cancer metastasis.	/	(64)
Breast cancer	PTX3 is strongly expressed in bone metastases of breast cancer, closely related to the transformation of epithelial cells with mesenchymal characteristics into breast osteoblast-like cells.	/	(65)
Breast cancer (promoting)	PTX3 is upregulated in bone metastases of breast cancer, correlated with poor survival while inducing cell migration, macrophage chemotaxis and osteoclast differentiation.	TNF-α/PTX3	(66)
Breast cancer	PTX3 is upregulated in aggressive breast cancer cell lines by bioinformatic analyses of public database.	/	(67)
Breast cancer (promoting)	The cancer stemness-promoting PTX3 is induced by PI3K activation in basal-like breast cancer cells through AKT- and NF-κB-dependent signaling.	PI3K/AKT/PTX3 and PI3K/NF-κB/PTX3	(69)
Breast cancer (promoting)	The overexpressed PTX3 in cancer cell promotes stemness, EMT, migration, and invasion, which is inhibited by rBmK AGAP through NF-κB and Wnt/β-catenin signaling pathway.	NF-κB/PTX3 and Wnt/β-catenin/PTX3	(70)
Breast cancer (promoting)	PTX3 is regulated by SH3RF3 through JNK-JUN pathway, thus enhancing breast cancer stem cell properties.	JNK/JUN/PTX3	(71)
Breast cancer	PTX3 is correlated with ER status in breast cancer at both mRNA and protein levels.	/	(72)
Prostate cancer (inhibitory)	PTX3 inhibits the activity of FGF2 or FGF8b, exerting anti-angiogenic and anti-neoplastic activity in prostate cancer.	PTX3/FGF2 and PTX3/FGF8b	(73)
Prostate cancer (inhibitory)	PTX3 inhibits dihydrotestosterone- or FGF8b-driven mitogenic activity in androgen-regulated human prostate cancer cells.	PTX3/FGF8b	(61)
Prostate cancer	PTX3 positive cells in prostate tumors are increased compared to benign lesions, and show an inverse correlation with the number of PD-L1 positive prostate cancer cells.	/	(74)
Prostate cancer	The C1q/PTX3 deposits without resulting in C5b-9 activation are extensively present in BPH patients who later develop into prostate cancer.	/	(75)
Prostate cancer	PTX3 levels are higher in the tumor lesions and serum of prostate cancer patients compared to those with prostatic inflammation/BPH, and increase progressively over time.	/	(76)
Prostate cancer	PTX3 is upregulated in prostate cancers at both mRNA and protein levels, and is positively correlated with expression of ERα and EGFR.	/	(77)
Prostate cancer	The PTX3 is highly expressed in high-grade prostate cancer lesions, and is positively correlated with ^18^F-choline uptake.	/	(78)
Prostate cancer	A strong PTX3 expression is observed in tumor samples of prostate cancer patients with bone metastases, compared to those without bone metastases or without cancer.	/	(79)
Lung cancer (promoting)	Deglycosylation of PTX3 by tunicamycin inhibits growth and migration of lung cancer cells, and sensitizes lung cancer to cisplatin through AKT/NF-κB signaling inactivation.	PTX3/AKT/NF-κB	(80)
Lung cancer (promoting)	Peripheral PTX3, correlated with shorter OS, is elevated in lung cancer *via* AKT/NF-κB signaling, while its silencing overcomes cisplatin resistance in NSCLC cells.	AKT/NF-κB/PTX3	(81)
Lung cancer (promoting)	Gemcitabine induces PTX3 upregulation *via* NF-κB signaling pathway, leading to augmented invasiveness of lung cancer cell, which is inhibited by Rg3.	NF-κB/PTX3	(82)
Lung cancer	PTX3 is significantly higher in squamous cell carcinoma cell lines, as well as in the serum of lung cancer patients compared to healthy individuals.	/	(83)
Lung cancer	Peripheral PTX3 levels are higher in COPD patients than in lung cancer patients, while in lung cancer patients with COPD, an elevated PTX3 level is associated with worse PFS.	/	(84)
Lung cancer	The level of PTX3 in bronchoalveolar lavage fluid is upregulated in lung cancer patients, especially in SCLC patients or lung cancer patients with obstructive pneumonia.	/	(85)
Lung cancer	Serum PTX3 serves as a novel and informative diagnostic biomarker for lung cancer.	/	(86)
Lung cancer	Serum PTX3 is higher in lung cancer patients than in heavy smokers, whereas high local PTX3 expression in tumors independently correlates with shorter survival.	/	(87)
Lung cancer	Elevated local PTX3 expression is correlated with reduced OS and DFS, and is an independent negative prognostic factor for OS in SCLC.	/	(88)
Colorectal cancer (inhibitory)	The promoter region and the CpG island of *PTX3* are highly methylated in CRC, leading to *PTX3* silencing, cancer development and growth.	/	(30)
Colorectal cancer (inhibitory)	PTX3 is regulated by two enhancers in CRC: enhancer 1 is epigenetically silenced by STAT3 in early stages, whereas enhancer 2 undergoes stage-dependent DNA methylation during CRC progression.	STAT3/PTX3	(32)
Colorectal cancer (inhibitory)	CRC cells obtain metastatic potential through methylation of the oncosuppressor PTX3.	/	(89)
Colorectal cancer	The high levels of circulating PTX3 combined with IL-8 and VEGF are associated with an increased risk of disease recurrence and a worse survival in CRC patients.	/	(90)
Colorectal cancer	Plasma PTX3 levels in CRC patients are higher than healthy controls, but decline after surgical resection, and can be elevated at the time of relapse.	/	(91)
Colorectal cancer	A high level of baseline serum PTX3 can independently predict a poor prognosis for CRC patients after curative resection.	/	(92)
Esophageal cancer (inhibitory)	PTX3 overexpression in ESCC cells reduces cellular proliferation and colony formation, increases the rate of apoptosis, and forms significantly smaller tumors *in vivo*.	/	(93)
Esophageal cancer	The inhibitory effect of JDTYS on esophageal precancerous lesions is related to upregulation of plasma PTX3.	/	(94)
Esophageal cancer	Peripheral PTX3 is induced after protocatechuic acid treatment in esophageal cancer, which may account for the decrease in high grade dysplasia.	/	(95)
Esophageal cancer	PTX3 shows differential methylation among ESCC lesions, remote normal tissues, and healthy controls, suggesting its potential as a diagnostic biomarker for ESCC.	/	(96)
Esophageal cancer	A majority of ESCC cell lines and tumor samples show down-regulated PTX3 expression due to gene promoter hypermethylation.	/	(29)
Liver cancer (promoting)	PTX3 is elevated in HCC specimens, promoting proliferation, migration, invasion, and EMT phenotype of cancer cells, and independently predicting poor prognosis of HCC patients.	/	(97)
Liver cancer	PTX3 levels show a progressive increase from healthy controls to HBV-infected patients and to HCC, identifying it as an independent risk factor with the potential to diagnose HCC in patients with chronic HBV infection.	/	(98)
Liver cancer	HCC patients present higher plasma PTX3 levels than patients with fibrosis, and the release of PTX3 in HCV infection can increase the risk of HCC occurrence.	/	(99)
Liver cancer	Circulating PTX3 shows no significant association with HCC disease severity, nor does it demonstrate diagnostic value.	/	(100)
Gastric cancer (promoting)	PTX3, elevated by BDNF/TrkB axis, is related to bone metastatic status of gastric cancer by enhancing gastric cancer-osteoblastic niche interactions.	BDNF/TrkB/PTX3	(101)
Gastric cancer (promoting)	PTX3 expression is elevated in advanced gastric cancer samples *via* TNF-α/NF-κB activation, thus promoting cancer cell migration and the recruitment of macrophages.	TNF-α/NF-κB/PTX3	(102)
Gastric cancer (inhibitory)	PTX3 is less expressed in gastric cancer, but overexpression of PTX3 inhibits gastric cancer cell migration, invasion, and EMT, which can be reversed by TNF-α treatment.	TNF-α/PTX3	(103)
Cervical cancer (promoting)	PTX3 is highly expressed in human cervical cancers, and contributes to tumorigenesis and metastasis of cervical cancer cells.	/	(104)
Cervical cancer (inhibitory)	The overexpressed miR-224 promotes the proliferation, migration, and invasion of cervical cancer cells by suppressing its downstream target PTX3.	miR-224/PTX3	(105)
Cervical cancer	A panel of 11 plasma proteins, including PTX3, can differentiate cervical cancer patients from healthy controls with high sensitivity and specificity.	/	(106)
Melanoma (inhibitory)	PTX3 inhibits FGF/FGFR-driven EMT in melanoma cells, repressing the tumorigenic and metastatic potential of melanoma cells.	PTX3/FGF2/FGFR1	(107)
Melanoma (inhibitory)	PTX3 overexpression suppresses the proliferation and migration of melanoma cells through FGF trapping approach.	/	(108)
Melanoma (promoting)	Melanoma-derived PTX3 promotes cancer cell invasion and migration *via* TLR4/NF-κB signaling pathway.	PTX3/TLR4/NF-κB	(109)

FGF, fibroblast growth factor; TNF-α, tumor necrosis factor-α; PI3K, phosphoinositide 3-kinase; AKT, protein kinase B; NF-κB, nuclear factor kappa-B; EMT, epithelial-mesenchymal transition; rBmK, recombinant *Buthus martensii Karsch*; AGAP, antitumor-analgesic peptide; Wnt, wingless-related integration site; SH3RF3, SH3 domain containing ring finger 3; JNK, c-Jun N-terminal kinase; ER, estrogen receptor; PD-L1, programmed death ligand 1; BPH, benign prostatic hyperplasia; EGFR, epidermal growth factor receptor; OS, overall survival; NSCLC, non-small cell lung cancer; COPD, chronic obstructive pulmonary disease; PFS, progression-free survival; SCLC, small cell lung cancer; DFS, disease-free survival; CRC, colorectal cancer; STAT3, signal transducer and activator of transcription 3; IL-8, interleukin-8; VEGF, vascular endothelial growth factor; ESCC, esophageal squamous cell carcinoma; JDTYS, Jie Du Tong Ye San; HCC, hepatocellular carcinoma; HBV, hepatitis B virus; HCV, hepatitis C virus; BDNF, brain-derived neurotrophic factor; TrkB, tropomyosin receptor kinase B; miR, microRNA; TLR4, Toll-like receptor 4.

### Breast cancer

Recent studies indicate that PTX3 inhibits FGF2-dependent (60) and FGF8b-induced (61) angiogenesis during breast cancer development. Bevacizumab, an anti-vascular endothelial growth factor antibody used in breast cancer treatment (62), was found to elevate peripheral PTX3 levels in patients (63). This finding suggests that therapeutic strategies targeting PTX3 could potentially enhance anti-angiogenic effects, particularly in contexts where FGF2-driven angiogenesis predominates.

Conversely, doxorubicin-induced PTX3 has been shown to positively regulate breast cancer metastasis (64). Furthermore, studies by Scimeca M et al. and Choi B et al. highlight PTX3’s role in bone-metastatic breast cancer (65, 66). Scimeca et al. identified elevated PTX3 levels in both the breast tumor microenvironment (TME) and bone microenvironment, where it facilitates the transition of epithelial cells with mesenchymal features into breast osteoblast-like cells, enhancing tumor aggressiveness (65). Choi et al. confirmed PTX3’s promotion of breast cancer cell migration and osteoclast differentiation (66). Additionally, *PTX3* is a differentially expressed gene in aggressive breast cancer cell lines (67). Notably, PTX3 upregulation is closely linked to breast cancer stem cell-like traits, which confer self-renewal and differentiation capacities, contributing to tumor progression, recurrence, and drug resistance (68). Collectively, in breast cancer, PTX3 overexpression can promote stem-like properties through various signaling pathways, including NF-κB, Wnt/β-catenin, AKT, and JNK (69–71).

Despite the apparent clarity of PTX3’s metastasis-promoting effects in breast cancer, the complex role of peripheral PTX3 requires further investigation, as it is closely associated with estrogen receptor (ER) status in breast cancer at both the mRNA and protein levels (72). Future research should focus on evaluating the prognostic value of PTX3 in breast cancer and developing targeted therapies aimed at this molecule.

### Prostate cancer

PTX3 may act as an antitumor agent in prostate cancer by targeting FGF2 and FGF8b to exert antiangiogenic and antineoplastic effects, as previously discussed (61, 73). However, recent studies by Scimeca et al. (74) explored the relationship between PTX3 and programmed death ligand 1 (PD-L1), a prominent target in cancer immunotherapy. They found that PTX3 was overexpressed in prostate cancer compared to benign lesions, suggesting its potential role in tumor progression. Additionally, an inverse correlation was observed between the number of PD-L1-positive cells and PTX3 expression in prostate cancer cells, indicating the need for further research to understand the interplay between PTX3 and PD-L1 in developing effective prostate cancer management strategies.

A single-center cohort study revealed PTX3 and C1q deposits in prostate cancer samples, which were absent in benign prostatic hyperplasia (BPH) (75). However, the deposited C1q did not activate the terminal complement pathway, specifically the formation of the MAC. Furthermore, the complement inhibitor CD59 was significantly upregulated in prostate cancer. In essence, this study suggests that PTX3, a potential biomarker for distinguishing prostate cancer from BPH, promotes tumor progression by inhibiting the activation of the terminal complement pathway through increased CD59 expression (75). Additionally, as early as 2014, the same group validated that elevated PTX3 expression could serve as a biomarker for predicting the progression from prostatic inflammation or BPH to prostate cancer (76), consistent with the findings of Ravenna L et al. (77).

Additionally, Urbano et al. identified prostate osteoblast-like cells (POLCs) in prostate cancer, which share similarities with breast osteoblast-like cells, particularly in terms of bone-related biomarkers like PTX3 (78). Their study further demonstrated that POLCs serve as a negative prognostic marker for prostate cancer bone metastasis, with PTX3 playing a significant role in the metastatic process, analogous to its involvement in breast cancer bone metastasis (79).

### Lung cancer

PTX3 has emerged as a potential effector in lung cancer treatment, particularly in combination with cisplatin. Specifically, both PTX3 deglycosylation and PTX3 knockdown *via* AKT/NF-κB signaling inhibition have been shown to suppress tumor growth, enhancing the susceptibility of lung cancer cells to cisplatin treatment (80, 81). Furthermore, the upregulation of PTX3 induced by gemcitabine treatment has been linked to increased aggressiveness of lung cancer cells through the NF-κB signaling pathway (82). These findings suggest that downregulating PTX3 could be a beneficial strategy for lung cancer therapy.

In addition, extensive research highlights the clinical relevance of PTX3 as both a diagnostic and prognostic marker in lung cancer. Early in 2009, Planque et al. conducted a proteomics analysis of conditioned media from four different lung cancer cell lines, identifying PTX3 among five novel candidates as a key biomarker. Subsequent validation confirmed that serum PTX3 levels are significantly elevated in lung cancer patients compared to healthy individuals (83), but still lower than in patients with chronic obstructive pulmonary disease (COPD) (84). Notably, the upregulated PTX3 levels in bronchoalveolar lavage fluid also serve as a biomarker for lung cancer, particularly in small cell lung cancer (SCLC) and in cases with obstructive pneumonia (85). Moreover, PTX3 has been identified as an effective marker to differentiate between lung cancer patients and individuals at high risk for the disease (86). Additionally, research by Infante et al. reinforced the diagnostic potential of serum PTX3 in lung cancer and demonstrated that high interstitial PTX3 expression in resected tumor specimens correlates with poor prognosis, indicating shorter survival (87). Elevated PTX3 expression was also associated with reduced overall survival (OS) and aggressive behavior in SCLC patients (88). These findings suggest that circulating PTX3 could be a valuable clinical tool, particularly in the management of SCLC patients.

### Colorectal cancer

In 2015 and 2017, two studies demonstrated the tumor-suppressive role of PTX3 in CRC, revealing some common findings. In the first study, the Alberto lab discovered that *PTX3* could be silenced by methylation of its promoter region and in a CpG rich putative enhancer encompassing exon 2 (30). This silencing triggered complement-mediated, macrophage-dependent and tumor-related inflammation, fostering a tumor-promoting environment and increasing susceptibility to CRC development and progression. In 2017, they further identified two enhancers of *PTX3*, whose methylation inactivated *PTX3*, counteracting its tumor-suppressive function in CRC (32). Additionally, they found that STAT3 was responsible for silencing *PTX3* through DNA methylation of enhancer 1 during the early stages of CRC progression in a stage-specific manner. Tsuji et al. also observed that metastatic colorectal carcinomas, including CRC liver metastases, were partially attributed to aberrant methylation of the oncosuppressor *PTX3* (89).

In addition to studies on *PTX3* methylation in CRC, growing research has focused on its prognostic value. In 2016, two studies simultaneously reported that upregulated postsurgical serum PTX3 was linked to CRC relapse (90, 91), and a subsequent cohort study confirmed the prognostic value of PTX3 as an independent indicator of poor prognosis in CRC patients following surgery (92). All three studies indicated that elevated peripheral PTX3 levels were associated with poorer prognosis in CRC patients.

### Esophageal cancer

Beyond CRC, a study also highlighted the tumor-suppressive role of PTX3 in ESCC (93). The researchers showed that PTX3 overexpression inhibited ESCC cell proliferation and colony formation while sensitizing them to apoptosis. Furthermore, two studies demonstrated that anthocyanins, found in the traditional Chinese herbal formula Jie Du Tong Ye San and black raspberries, as well as a major anthocyanin metabolite, protocatechuic acid, could upregulate PTX3 expression and exert protective effects in esophageal carcinoma (94, 95).

A two-stage molecular epidemiological study by Peng et al. explored DNA methylation profiles for biomarker discovery in ESCC among Chinese populations (96). Their findings confirmed the diagnostic potential of aberrant *PTX3* gene methylation in ESCC patients. Furthermore, they demonstrated that PTX3 downregulation resulted from promoter hypermethylation in ESCC, suggesting that PTX3 could serve as a biomarker for the disease (29).

### Liver cancer

In hepatocellular carcinoma (HCC), PTX3 has been implicated in tumor metastasis and poor prognosis (97). Both *in vitro* and *in vivo* studies demonstrated that PTX3 overexpression promotes HCC cell proliferation, migration, and invasion by inducing epithelial-mesenchymal transition (EMT). Furthermore, a strong association between elevated PTX3 expression and higher serum alpha fetoprotein (AFP) levels, larger tumor size, and more advanced tumor stages suggests that PTX3 could serve as a prognostic biomarker for HCC (97). Additionally, serum PTX3, an independent risk factor for HCC, may be used as a diagnostic marker, particularly for distinguishing early-stage HCC in hepatitis B virus (HBV)-infected patients, especially those who are AFP-negative (98). In the context of HCV infection, PTX3 has been identified as a risk factor for hepatocarcinogenesis, with increased PTX3 levels correlating with a higher risk of developing HCC in HCV-infected patients (99). However, a contrasting study challenged these findings, asserting that PTX3 is not linked to disease severity in HCC patients and does not serve as a diagnostic marker for HCC (100). Consequently, the role of PTX3 in HCC remains unclear and warrants further investigation.

### Gastric cancer

In gastric cancer, PTX3 exhibits an opposing effect. Choi et al. reported that increased PTX3 expression, driven by the brain-derived neurotrophic factor (BDNF)/tropomyosin receptor kinase B (TrkB) axis, enhances interactions between gastric cancer cells and the osteoblastic niche, thus promoting gastric cancer bone metastasis (101). Another study reinforced PTX3’s role in promoting migration of gastric cancer cells and identified its ability to recruit macrophages through TNF-α/NF-κB activation-driven PTX3 upregulation (102). Silencing PTX3, in turn, reduced gastric cancer-related inflammation. However, Cui et al. observed that TNF-α could mediate gastric cancer progression, including invasion and migration, by downregulating PTX3, and that overexpression of PTX3 could reverse these effects both *in vitro* and *in vivo* (103). These divergent findings suggest that the impact of PTX3 on gastric cancer may depend on the underlying molecular mechanisms and the characteristics of different cell lines, necessitating further research to clarify its precise role in this context.

### Cervical cancer

In human cervical cancer cells, PTX3 knockdown has been shown to inhibit tumorigenicity and metastasis, particularly lung metastasis (104). In another study, miR-224 was found to be associated with PTX3 expression in cervical cancer (105). According to their findings, miR-224 is overexpressed while PTX3 expression is reduced, promoting cell proliferation, migration, and invasion in cervical carcinoma. Furthermore, *PTX3* is a target gene of miR-224 and is downregulated by it. Upregulating PTX3 by inhibiting miR-224 may help protect against cervical cancer development (105). Notably, PTX3 is also considered a potential innovative diagnostic biomarker for cervical cancer (106). Given that the exact role of PTX3 in cervical cancer remains unclear, further research focusing on PTX3’s interactions with signaling pathways, microRNAs, and other molecular components is necessary.

### Melanoma

In melanoma, PTX3 has been shown to inhibit FGF2-dependent EMT, thereby reducing cell proliferation, carcinogenesis, and metastasis (107). In 2019, it was reconfirmed that PTX3, as a natural FGF trap inhibitor, could significantly suppress melanoma tumor growth and liver metastasis, thus inhibiting the proliferation and migration of melanoma cells (108), making PTX3 a promising therapeutic target for melanoma patients. However, apart from its effects on FGF signaling, autocrine PTX3 in melanoma has been linked to the TLR4/NF-κB signaling pathway, where it exerts opposing effects (109). Under these conditions, PTX3 secreted by melanoma cells promotes tumor migration, invasion, and the expression of EMT factors (109). Therefore, the role of PTX3 in melanoma remains ambiguous, influenced by the different signaling pathways it engages.

## Other functions of PTX3

### Inflammation

PTX3 plays a pivotal role in the inflammatory response. During inflammation, peripheral PTX3 levels rise significantly, primarily stored in neutrophils and rapidly released upon activation (110). PTX3 is synthesized by a variety of cells, including those of the myeloid lineage, vascular and lymphatic endothelial cells, smooth muscle cells, fibroblasts, epithelial cells, adipocytes, mesangial cells, astrocytes, and microglia (111). PTX3 possesses antibody-like properties and functions as a fluid-phase PRM (112). It interacts with different complement components through the classical, alternative, and lectin pathways (6). To maintain a balance between inadequate immune response and excessive inflammatory damage, PTX3 has been shown to regulate early neutrophil recruitment through a negative feedback mechanism by inhibiting P-selectin (113, 114). Additionally, PTX3 may modulate the phagocytosis of apoptotic cells, further helping to control the inflammatory response (6).

Inflammation has long been recognized as a key factor in tumor progression (115). Among the studies mentioned, a significantly higher level of PTX3 was found in patients with COPD compared to lung cancer patients. Furthermore, elevated PTX3 levels in lung cancer patients with COPD were associated with worse progression-free survival (PFS) (84). This was further corroborated by findings showing upregulated PTX3 levels in bronchoalveolar lavage fluid from lung cancer patients, particularly those with SCLC or obstructive pneumonia (85). Both studies highlight the role of PTX3, an inflammatory factor, as a potential biomarker in lung cancer. Similarly, compared to patients with prostatic inflammation or BPH, prostate cancer patients exhibited elevated peripheral PTX3 levels, which increased over time, suggesting a tumor-promoting role of PTX3 during prostatic inflammation or BPH (75, 76). Collectively, these findings suggest that PTX3 may play an active role in the inflammation-to-cancer transition, warranting further experimental investigation.

### Infection

PTX3 is involved in innate humoral immunity, particularly in defending against specific pathogens. Several studies have highlighted its contribution to resisting infections. For example, *Ptx3^-/-^* mice exhibit impaired recognition of conidia by alveolar macrophages and dendritic cells, making them more susceptible to invasive pulmonary aspergillosis compared to wild-type mice (19). PTX3 also enhances neutrophil-mediated phagocytosis of microorganisms through opsonic activity, utilizing FcγRII-, CD11b-, and complement-dependent mechanisms (6). Additionally, PTX3 has a specific binding site that interacts with macrophage receptors, especially FcγRIII/CD16 and FcγRII/CD32, with dissociation constants (KD) of 1.6μM and 18.7μM, respectively (111). Overall, PTX3 plays a significant role in anti-infection by enhancing pathogen recognition, facilitating pathogen uptake by phagocytes, and activating complement cascades (6).

PTX3 has been shown to recognize microbial components and specific microorganisms during infection (6, 112). A wide range of bacteria, fungi, and viruses can bind specifically to PTX3, including *Aspergillus fumigatus* conidia, zymosan, various gram-positive and gram-negative bacteria, and human and murine cytomegalovirus, as well as influenza A virus (IVA) (111). In urinary tract infection (UTI) mouse models, urothelium invaded by uropathogenic *Escherichia coli* (UPEC) produces PTX3 *via* a TLR4- and MyD88-dependent pathway. PTX3 binding to UPEC accelerates phagocyte maturation and enhances neutrophil phagocytosis. Elevated PTX3 levels in serum and urine correlate with the severity of UTI in patients (116). Human breast milk, which contains high concentrations of PTX3, is primarily produced by mammary epithelial cells and CD11b^+^ milk cells. Oral administration of Ptx3 has been shown to protect neonatal mice from *Pseudomonas aeruginosa* pulmonary infection, highlighting its vital role in protecting newborns with immature immune systems from infections (117). However, PTX3 also exhibits a dual function in infections. While it helps combat infections, it can also promote the invasion and replication of arthritogenic alphavirus, extending disease duration (118).

During the COVID-19 pandemic, emerging evidence highlighted the association between PTX3 and COVID-19 infection. Serum PTX3 levels were highest in intensive care unit (ICU)-admitted COVID-19 patients compared to non-ICU patients and healthy controls, suggesting a link to disease severity (119). PTX3 has also been proposed as an early predictive biomarker for co-infections (such as fungal or bacterial secondary infections) in COVID-19 patients (120). A meta-analysis identified PTX3 as a reliable marker for poor outcomes following COVID-19 infection (121), and it was found to be a useful clinical biomarker for predicting the 30-day risk of respiratory failure and mortality in COVID-19 patients, regardless of remdesivir or dexamethasone treatment (122). However, another study denied any association between PTX3 and long COVID-19 (persistent symptoms 6-12 months post-infection), while reinforcing its role in acute COVID-19 infection (123).

### Tissue remodeling

PTX3 plays a critical role in innate immunity, particularly in tissue repair following injury (124). In various mouse models of non-infectious tissue damage, *Ptx3* deficiency is linked to fibrin deposition and persistence, leading to increased collagen accumulation and an altered thrombotic response to injury (5, 125). PTX3 interacts with fibrinogen/fibrin and plasminogen (Plg), promoting pericellular fibrinolysis, thereby facilitating tissue repair and remodeling (126). *Ptx3*-deficient mice with skin wounds exhibit impaired healing, characterized by excessive clotting, fibrin accumulation, and delayed reepithelialization during the early stages of wound healing (125). These findings suggest that the absence of PTX3 disrupts normal pericellular fibrinolysis, hindering tissue remodeling. Additionally, PTX3 deficiency is associated with increased thrombosis risk and reduced blood flow velocity (125). PTX3 also plays a synergistic role in preventing thrombosis, potentially by targeting fibrinogen through its N-terminal domain and interacting with collagen *via* its C-terminal domain (127).

Hyaluronan (HA) and its primary receptor CD44 are key regulators of bone homeostasis. Notably, several studies have shown that PTX3 promotes bone regeneration. In the context of periodontitis, Ptx3 was found in the HA-dependent pericellular matrix (PCM) of mouse pre-osteoblast cells, where it enhances HA synthesis and Cd44 expression, thereby strengthening the HA-Cd44 interaction. This interaction activates the focal adhesion kinase (FAK)/AKT signaling cascade, which further upregulates the expression of HA synthases (1/2/3) and Cd44, establishing a positive feedback loop initiated by Ptx3 (128). Consistent with these findings, Ptx3-assembled HA-rich PCM promotes chondrocyte matrix synthesis and maturation through the same CD44/FAK/AKT signaling, facilitating endochondral ossification during fracture healing and traumatic heterotopic ossification in mice (129).

### Female reproductive system

PTX3 is crucial for female fertility, serving as a structural component of the cumulus oophorus extracellular matrix (130), which is synthesized by the cells surrounding the oocyte prior to ovulation (131). When premixed with inter-α-inhibitor (IαI) and TNF-stimulated gene-6 (TSG-6), PTX3 can be incorporated into the HA film, an essential component of the cumulus matrix. A deficiency in any of these molecules can lead to sterility (132, 133). Furthermore, the interaction between the heavy chain (HC) and PTX3 in the cumulus matrix helps protect the reproductive tract by modulating leukocyte activity and creating an optimal microenvironment for fertilization (133). PTX3 also influences female infertility conditions, such as polycystic ovary syndrome (PCOS), a chronic inflammatory disorder characterized by hyperandrogenism, persistent anovulation, and polycystic ovarian changes (134). PTX3 is primarily produced at sites of local inflammation; however, in PCOS follicles, its concentration is significantly lower than in normal ovulatory follicles, which may be linked to hyperandrogenemia and a higher luteinizing hormone (LH)/follicle stimulating hormone (FSH) ratio (130). Thus, PTX3 plays a unique role in follicular development and the female reproductive microenvironment.

## Concluding remarks

Pentraxins, as part of PRMs, play a pivotal role in initiating the innate immune response (135). Despite differences in the length of the N-terminal region (**Figure 1**), PTX3 shares structural and functional similarities with CRP and SAP, all contributing to immune system activity (136). PTX3 binds to various molecules, inhibiting the complement system in some contexts, while the plastic-immobilized PTX3 accelerates this process by facilitating the LP (**Figure 2**) (23). As an integral part of innate immunity, PTX3 functions as a double-edged sword in infection and inflammation. In promoting inflammation, PTX3 participates in antigen recognition, complement system activation, and the recruitment of inflammatory cells. It also enhances viral adsorption and replication, thereby prolonging the infection. In contrast, PTX3 can inhibit inflammation by binding to antigen epitopes and regulating the phagocytosis of apoptotic cells. Given its complex relationship with inflammation, PTX3 has become a key indicator of inflammatory activity. Additionally, PTX3 interacts closely with neutrophils, macrophages, and mesenchymal stem cells (MSCs), playing a vital role in tissue remodeling. With the growing recognition that inflammation and tissue repair are closely linked to tumorigenesis, the relationship between PTX3 and cancer has been the subject of extensive research.

PTX3 exerts antiangiogenic and anticarcinogenic effects (**Figure 3**), primarily by inhibiting the FGF/FGFR pathway (137), as well as exerting tumor-suppressing or -promoting effects through other signaling pathways, including NF-κB and PI3K/AKT (**Table 1**) (69). Given its involvement in tumorigenesis-related pathways, PTX3’s relationship with cancer is complex. On one hand, PTX3 has been shown to inhibit angiogenesis and tumorigenesis in cancers such as breast, prostate, colorectal, esophageal, gastric, cervical, and melanoma. On the other hand, PTX3 promotes cancer initiation and metastasis in certain cancers, including liver cancer. It is particularly intriguing to consider whether *PTX3* is regulated by epigenetic mechanisms, such as DNA methylation or histone modification, as the expression levels of PTX3 can vary within a single cancer type, sometimes exerting opposing effects.

For example, in CRC, *PTX3* silencing due to promoter hypermethylation promoted cancer development and progression (30), while another study reported higher peripheral PTX3 levels in CRC patients compared to healthy controls (91). These discrepancies may be attributed to the diverse sources of PTX3. It might be possible that local silencing of PTX3 expression in CRC samples or cell lines contributes to tumor progression, while elevated peripheral PTX3 levels could be secreted by other cells, such as neutrophils and monocytes. This suggests a potential dichotomy between the local tumor site and the peripheral circulation. The opposite roles of PTX3 within a single cancer type may also be explained by the involvement of different signaling pathways. For instance, in breast cancer, PTX3 inhibits dihydrotestosterone- and FGF8b-driven cell proliferation (61), while elevated PTX3 levels, induced by TNF-α stimulation in a bone metastatic breast cancer cell line, promote cell migration, macrophage chemotaxis, and subsequent osteoclast differentiation (66). Similarly, contrasting roles for PTX3 have been observed in gastric cancer under TNF-α stimulation. One study emphasized PTX3’s inhibitory role in migration and invasion in gastric cancer cells (BGC-823 and SGC-7901) (103), while another study highlighted its role in promoting migration of the HTB135 cell line, a bone-metastatic human gastric cancer cell line (102). This discrepancy may arise from differences in the invasiveness of the cell lines used. The migration-promoting effect of PTX3 in bone-metastatic gastric cancer cells aligns with another study that demonstrated PTX3 upregulation *via* the BDNF/TrkB axis, enhancing gastric cancer-osteoblastic niche interactions and contributing to bone metastasis (101).

Given the widespread use of immunotherapy in cancer treatment and the critical role PTX3 plays in the immune system, there is significant potential for PTX3 to serve as a biomarker for predicting cancer prognosis. Moreover, the development of drugs targeting PTX3 appears promising, though no specific agonists or antagonists are currently available. Numerous studies have highlighted the role of *PTX3* promoter methylation in tumor progression (29, 30, 32, 96), suggesting that drugs targeting DNA methylation could help mitigate tumor progression, particularly in cancers of the digestive system. Furthermore, given PTX3’s pivotal role in inflammation and tissue repair, especially in bone remodeling, cancer patients with bone metastases may benefit from PTX3 inhibition, or even TNF-α inhibition (66, 102), an area that warrants further exploration.

While considerable efforts have been made to understand the structure and function of PTX3, identifying receptors beyond P-selectin that specifically bind PTX3 remains a key research objective. Investigating the dynamic changes in PTX3 within the tumor microenvironment may help uncover new insights. Additionally, it is important to consider the close relationship between PTX3 and microbes. PTX3 may be linked to the gut microbiota or act as a component of the mucosal immune barrier, potentially influencing tumor regulation. Therefore, a deeper understanding of PTX3’s role in tumorigenesis, as well as its potential in targeted therapy, diagnosis, and prognosis, is still needed. This review is expected to provide a solid foundation and help accelerate future research in these areas.
